# Supplementary material for: Cryo-EM structures of the pore-forming A subunit from the Yersinia entomophaga ABC toxin
Source: Nat Commun. 2019 Apr 26;10:1952. doi: 10.1038/s41467-019-09890-8 (PMC6486591; doi:10.1038/s41467-019-09890-8)
Supplement: Supplementary file 1 — Supplementary Information [file 41467_2019_9890_MOESM1_ESM.pdf]

Cryo-EM structures of the pore-forming A subunit from the *Yersinia entomophaga* ABC toxin

Piper et al.

Supplementary Figure 1

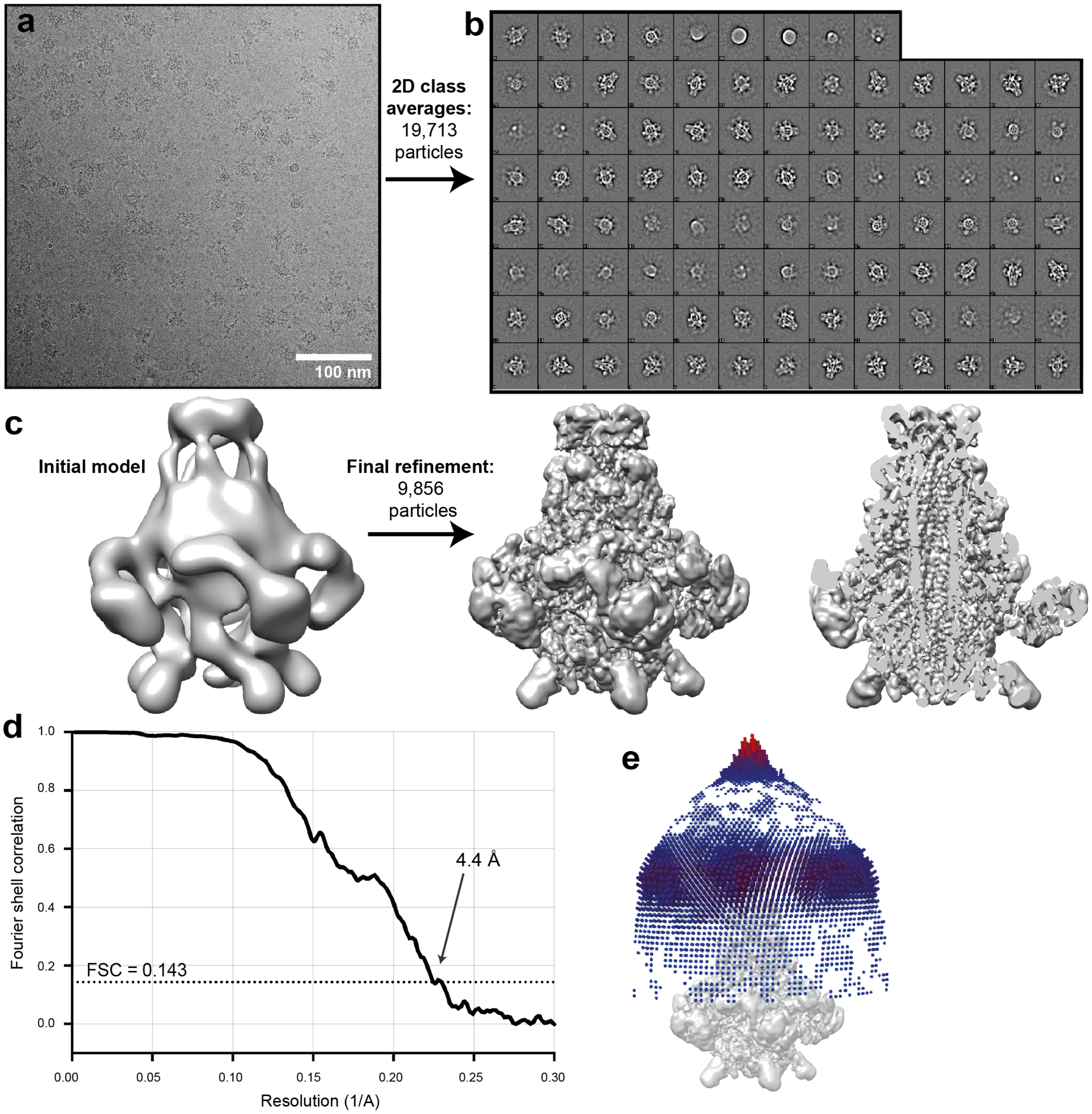

Supplementary Figure 1. Single particle analysis of YenTcA pre-pore form. (a) Representative micrograph of YenTcA pre-pore particles following dose-weighted motion-correction (scale bar: 100 nm). **(b)** 2D class averages representing ~19,000 picked particles generated in EMAN2. Note that class averages were generated using particles low pass filtered to 15 Å, as is standard in EMAN2 and so secondary structure features are not discernible. **(c)** Initial 3D reconstruction and subsequently refined 3D structure using ~10,000 "shiny" full resolution particle images. Both maps have C5 symmetry imposed. As for Fig. 1, the final map has been post-processed using a variable low pass filter according to local resolution. **(d)** Fourier shell correlation (FSC) indicates an estimated resolution of 4.4 Å according to the 0.143 criterion. **(e)** Angular distribution of particles contributing to the final map (red: high occurrence, blue: low occurrence).

Supplementary Figure 2

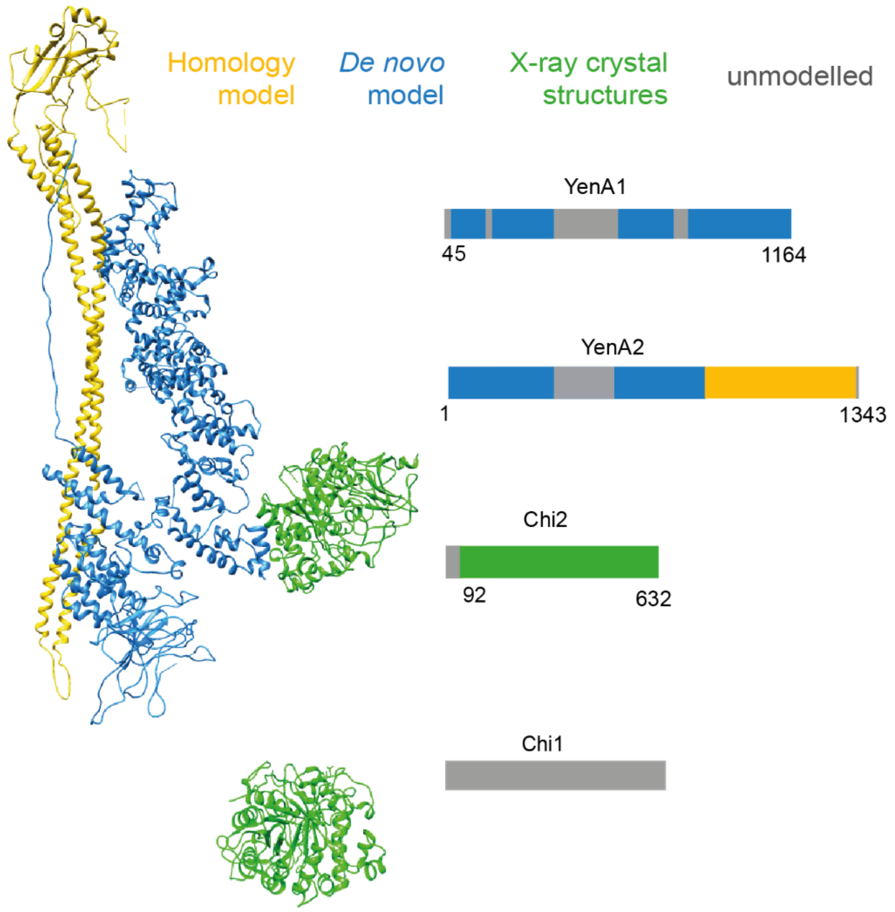

Supplementary Figure 2. Graphical overview of the model building strategy for YenTcA. A single YenTcA protomer is shown as a ribbon diagram, coloured according to the model building strategy employed. A homology model was built initially for the translocation pore and the TcB-binding domain (shown in gold) using the TcdA1 crystal structure (PDB ID: 4o9y) as a template. This was refined into the map density using the interactive molecular dynamics flexible fitting tools implemented in ISOLDE[1]. The majority of the YenA1 and YenA2 shell domains (shown in blue) were built *de novo* using ISOLDE, aided by the predominance of α-helical secondary structure in this region. Bulky side chains were also clearly visible in this region of the EM map and were used to guide the register of the polypeptide backbone. The existing crystal structure of Chi2 was fitted into the map (green) and then interactively refined into the map density, again using the molecular dynamics flexible fitting tools in ISOLDE. Finally, the refined protomer coordinates were copied to the symmetry related chains and the ensemble model was subject to a final molecular dynamics refinement under strict C5 symmetry using ISOLDE. The orientation of the Chi1 crystal structure within the map could not be unambiguously determined due to the poor quality of the map density in this region and so was excluded from the final refinement and statistical calculations.

Supplementary Figure 3

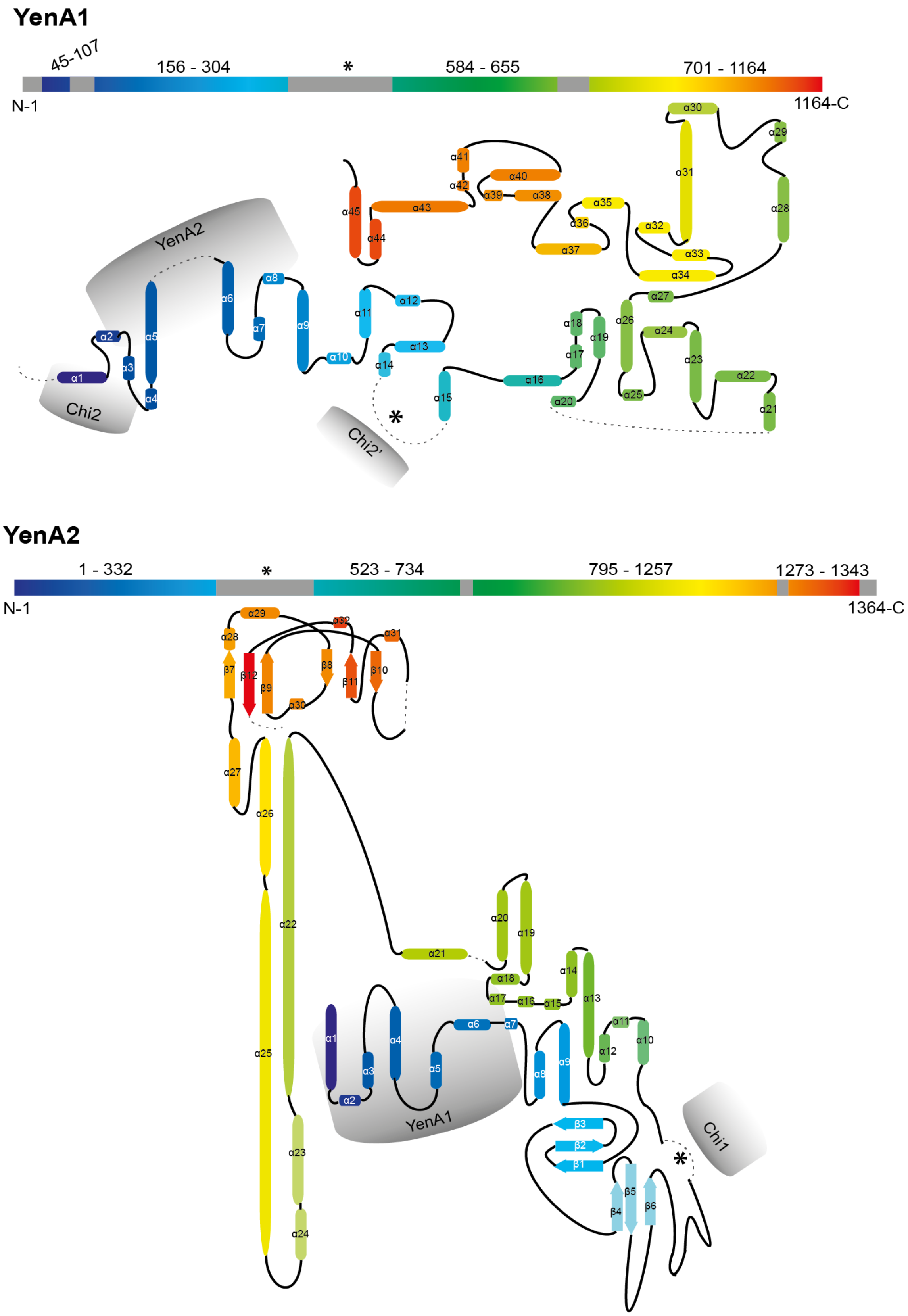

Supplementary Figure 3. Secondary structure topology of YenA1 and YenA2. Secondary structure is displayed as tubes (α-helix) and arrows (β-sheet) in rainbow colours from N- to C-terminus. Dotted lines indicate missing parts of the model. Grey areas within the topology model indicate surface interactions with the chains indicated. Asterisks (*) identify regions of high sequence variability, corresponding to putative receptor-binding domain insertion points.

Supplementary Figure 4

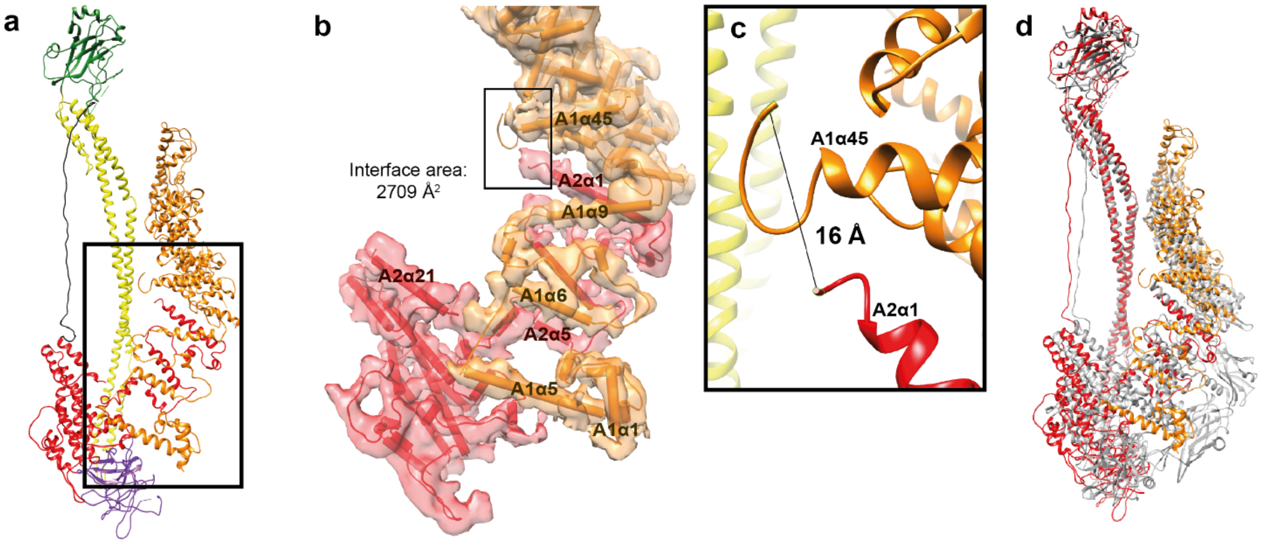

Supplementary Figure 4. YenA1 and YenA2 surface interactions. (a) Atomic model of a protomer of YenA1 (orange) and YenA2 (red, purple, black, yellow, green). **(b)** Secondary structure renderings of YenA1 (orange) and YenA2 (red) α-helical shell domains shown with the corresponding EM map density superimposed, highlighting the extensive interface formed by the interwoven α-helices. **(c)** The C-terminus of YenA1 and N-terminus of YenA2 are separated by a relatively short distance (~16 Å between the α-carbons of V1164 (YenA1 α45) and M1 (YenA2 α1)). **(d)** Structural overlay of the combined YenA1 (orange) and YenA2 (red) chains on the structure of *P. luminescens* TcdA1 (PDB ID: 4o9y). Alignment of the two structures was focused on the pore-forming helices.

Supplementary Figure 5

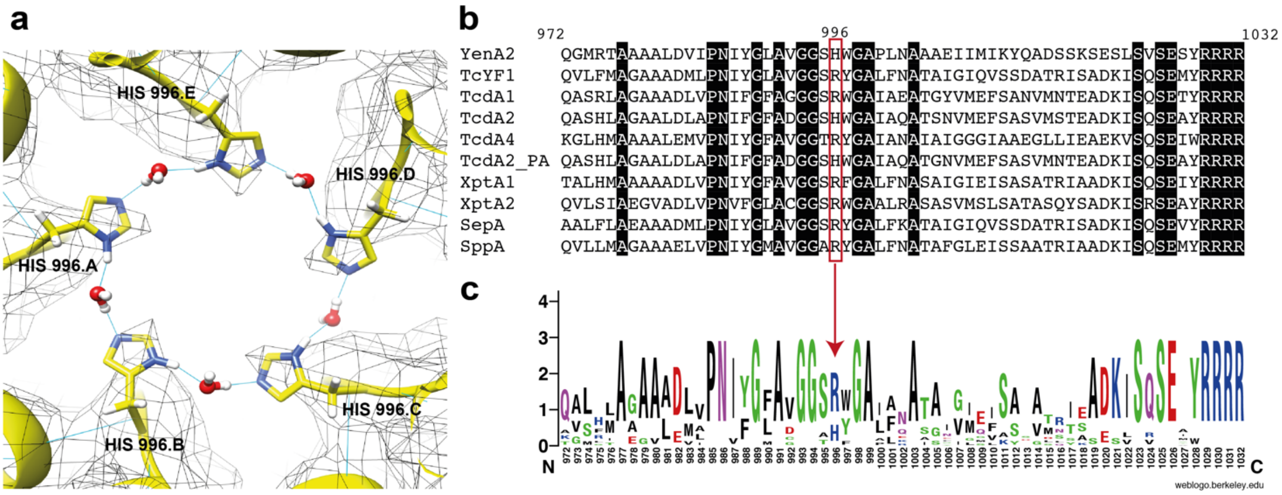

Supplementary Figure 5: Histidine 996 forms a non-conserved pH gate. (a) Molecular modelling of the pH gate of YenA2 at H996. The imdizaole groups of the histidine side chains appear well positioned to form a ring of stabilising H-bond interactions with bridging water molecules (cryo-EM map is shown as a mesh). This arrangement is predicted to be destabilised at lower pH (i.e. below the pKa of the histidine sidechain) where the imidazole group becomes doubly protonated. **(b)** Amino acid sequence alignment of the predicted lower pore region of ten TcA-like protein sequences. Completely conserved residues are highlighted in black and the position of H996 is enclosed in a red box. **(c)** Sequence logo summarising the sequence conservation observed in (b). The degree of conservation corresponds to the size of the respective amino acid one letter code. Of note is the poor conservation of H996, replaced by an arginine in most other TcA sequences (*Yersinia frederikseniii* TcYF1, *Photorhabdus luminescens* TcdA1 and TcdA4, *Xenorhabdus nematophilus* XptA1 and XptA2, *Serratia nematophila* SepA, *Serratia proteamaculans* SppA). Sequences in which the amino acid corresponding to H996 is a histidine are *Photorhabdus luminescens* TcdA2 and *Photorhabdus asymbiotica* TcdA2_PA.

Supplementary Figure 6

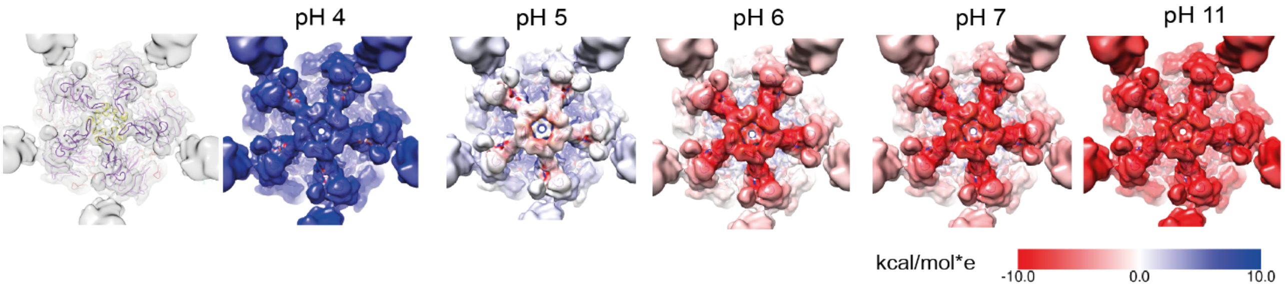

Supplementary Figure 6. Predicted effect of pH on the putative electrostatic lock. Electrostatic surface potential calculations simulated across a range of pHs predict that repulsive forces in the pore-closing loop predominate at all pHs but for a narrow window at pH 5.

Supplementary Figure 7

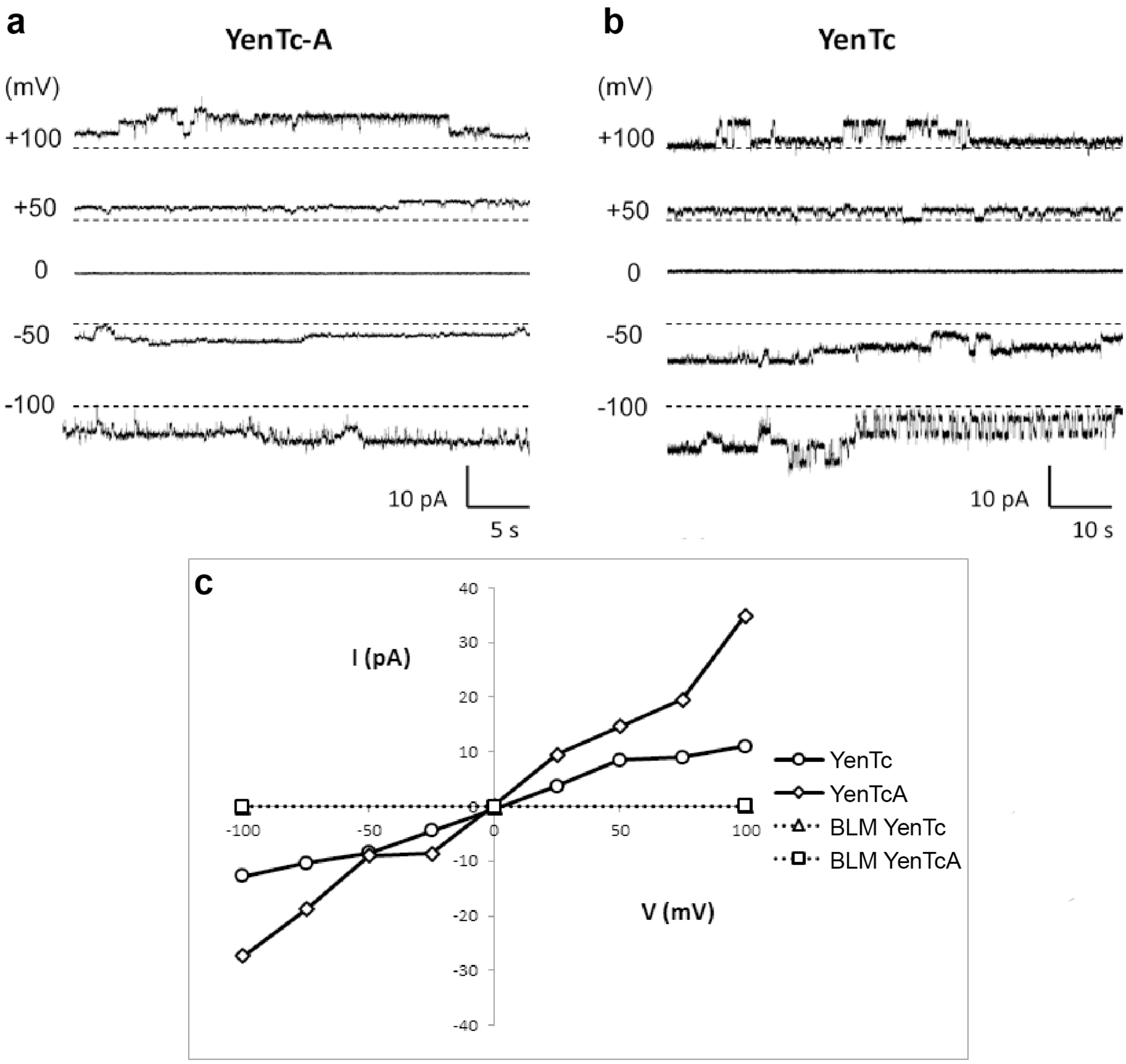

Supplementary Figure 7. Pore-forming properties of YenTc. (**a,b**) Representative channel recordings from black lipid membranes (BLMs) to which YenTcA or YenTc was added. Activity was seen in 18/20 (YenTc) or 5/6 (YenTcA) replicate experiments. Recordings were taken at applied voltages of 100 mV to -100 mV in 25 mV increments. Recordings at +/- 100 mV, +/- 50 mV and 0 mV are shown. Current responses reversed at 0 mV as expected. (**c**) Voltage/current dependency plots for YenTcA (diamonds) and YenTc (circles). The steeper slope of the YenTcA plot compared to the holotoxin (YenTc) was also reported in a comparable analysis of the *P. luminescens* TcdA1/PTC3 toxins[2] and is likely due to the attached BC subunit partly blocking the pore. Pre-control recordings (dashed lines) are virtually superimposable with the x-axis, indicating no detectable activity.

Supplementary Figure 8

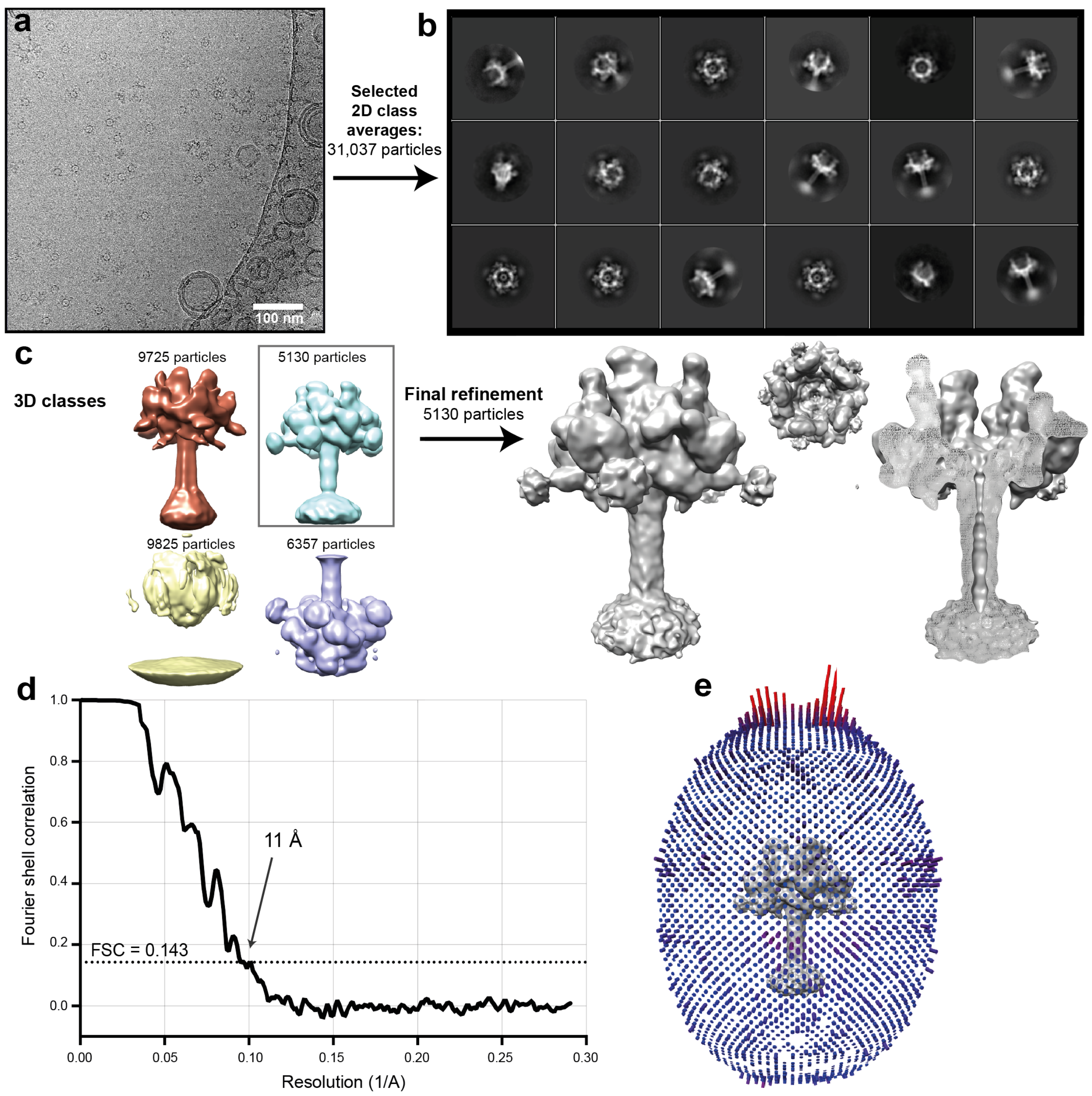

Supplementary Figure 8. Single particle analysis of YenTcA pore form in POPC liposomes. (a) Representative micrograph of YenTcA pore form particles following dose-weighted motion-correction (scale bar: 100 nm). **(b)** Selected 2D class averages for 3D classification. **(c)** 3D models obtained following 3D classification in Relion. Particles contributing to the turquoise 3D class were used for further processing to produce the final post-processed and masked map of the YenTcA pore form (grey). **(d)** Fourier shell correlation (FSC) indicates an estimated resolution of 11 Å according to the 0.143 criterion. **(e)** Angular distribution of particles contributing to the final refinement map (red: high occurrence, blue: low occurrence).

Supplementary Figure 9

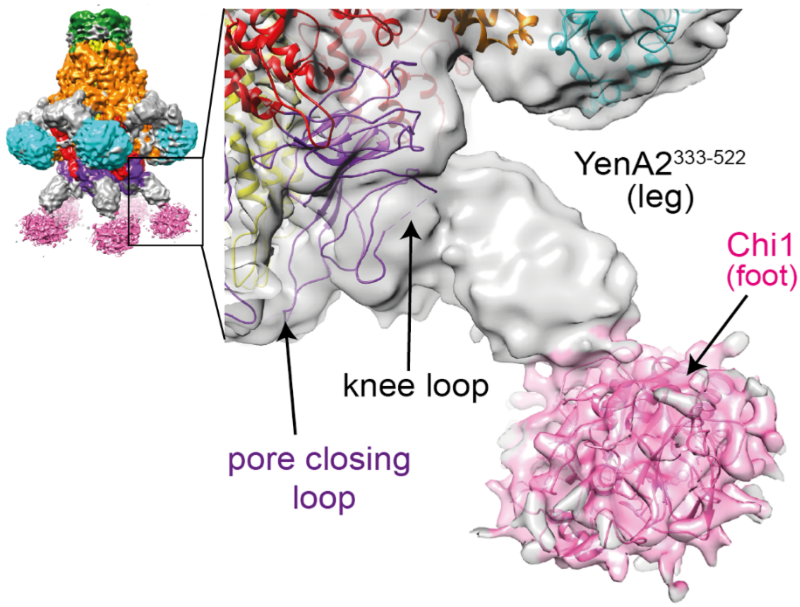

Supplementary Figure 9. Coupling of Chi1 to the pore-closing loop. The crystal structure of Chi1 (PDB ID: 3oa5) was docked as a rigid model into the "foot" density of YenTcA. Based on the size of Chi1, this is the only location where Chi1 could plausibly be fitted into the YenTcA map and despite the poor resolvability of this region, the overall fit of the structure to the EM map is reasonable. The grey density immediately adjacent to Chi1 (leg) is in turn immediately adjacent to the neuraminidase-like domain of YenA2. While the resolution of the leg is not sufficient to facilitate model building, residues 333-522 of YenA2, which represent a 188 amino acid insertion into the "knee loop" of the neuraminidase-like domain, are the only possible residues that could fill this space, consistent with the location of the flanking YenA2 sequences. The Chi1 feet of YenTc are thus coupled to the pore-closing loop within the neuraminidase like domain, via the YenA2 leg domain.

Supplementary Figure 10

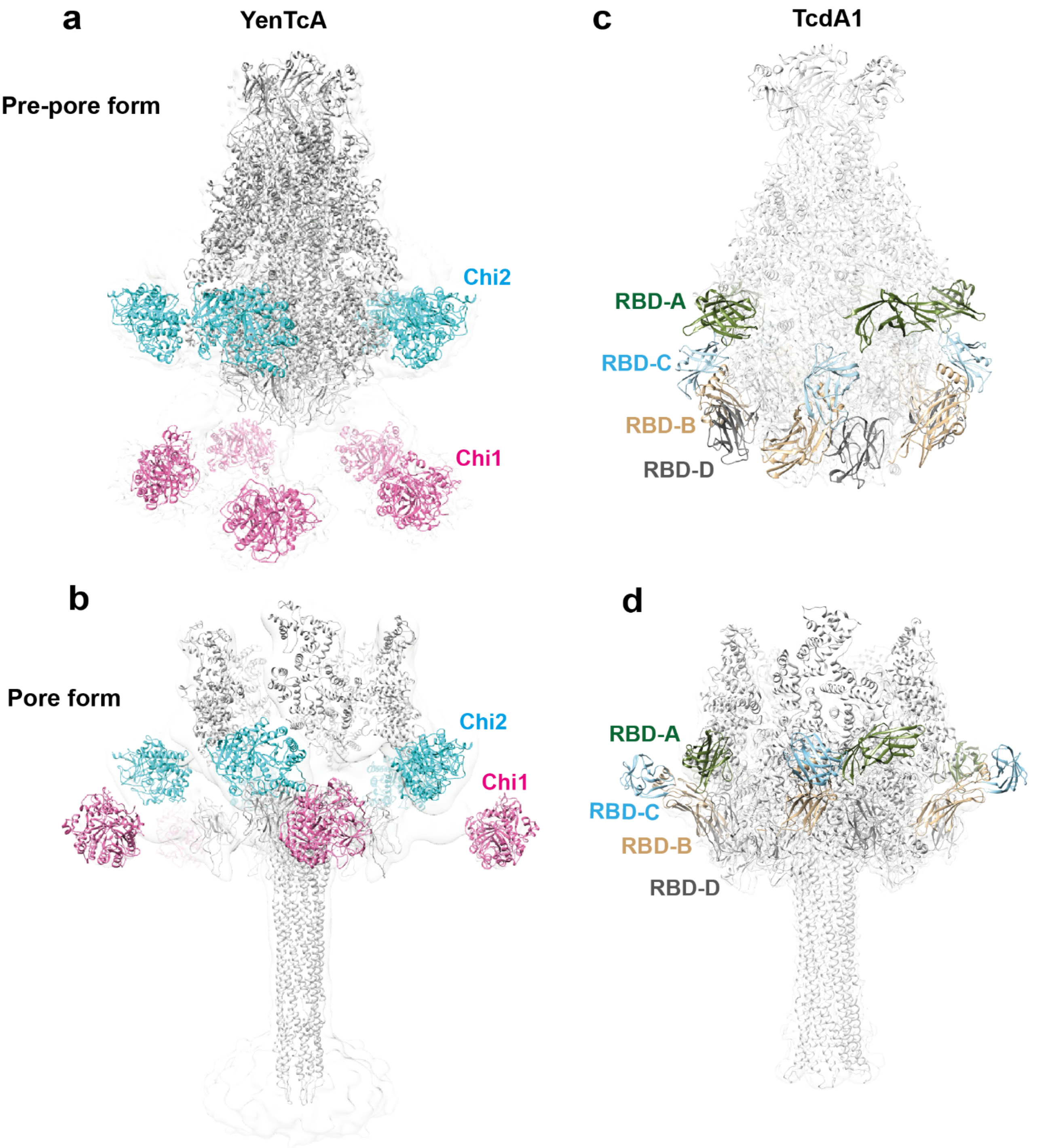

Supplementary Figure 10. Comparison of putative receptor-binding domains in TcdA1 and YenTcA. Atomic models of YenTcA (**a**) and TcdA1 (**b**) in the pre-pore form. Atomic models of the YenTcA (c) and TcdA1 (d) in the pore form. Note that the pore form structure of YenTcA was generated by rigid fitting of structural domains derived from the pre-pore structure and so the pore is not modelled in an open state. In all structures, putative receptor-binding domains are coloured to distinguish them from the α-helical shell and the translocation pore, which are both coloured in grey. Chi1 and Chi2 are coloured in pink and cyan, respectively. Putative receptor-binding domains (RBD) of TcdA1 are coloured in dark green (RBD-A), gold (RBD-B), light blue (RBD-C) and silver (RBD-D). RBD-B and RBD-C are inserted into a loop of the TcdA1 neuraminidase-like domain that overlays with the knee loop of the YenA2 neuraminidase-like domain. As shown in Supplementary Fig. 9, the interface for Chi1 involves residues inserted into the YenA2 knee loop.

Supplementary Figure 11

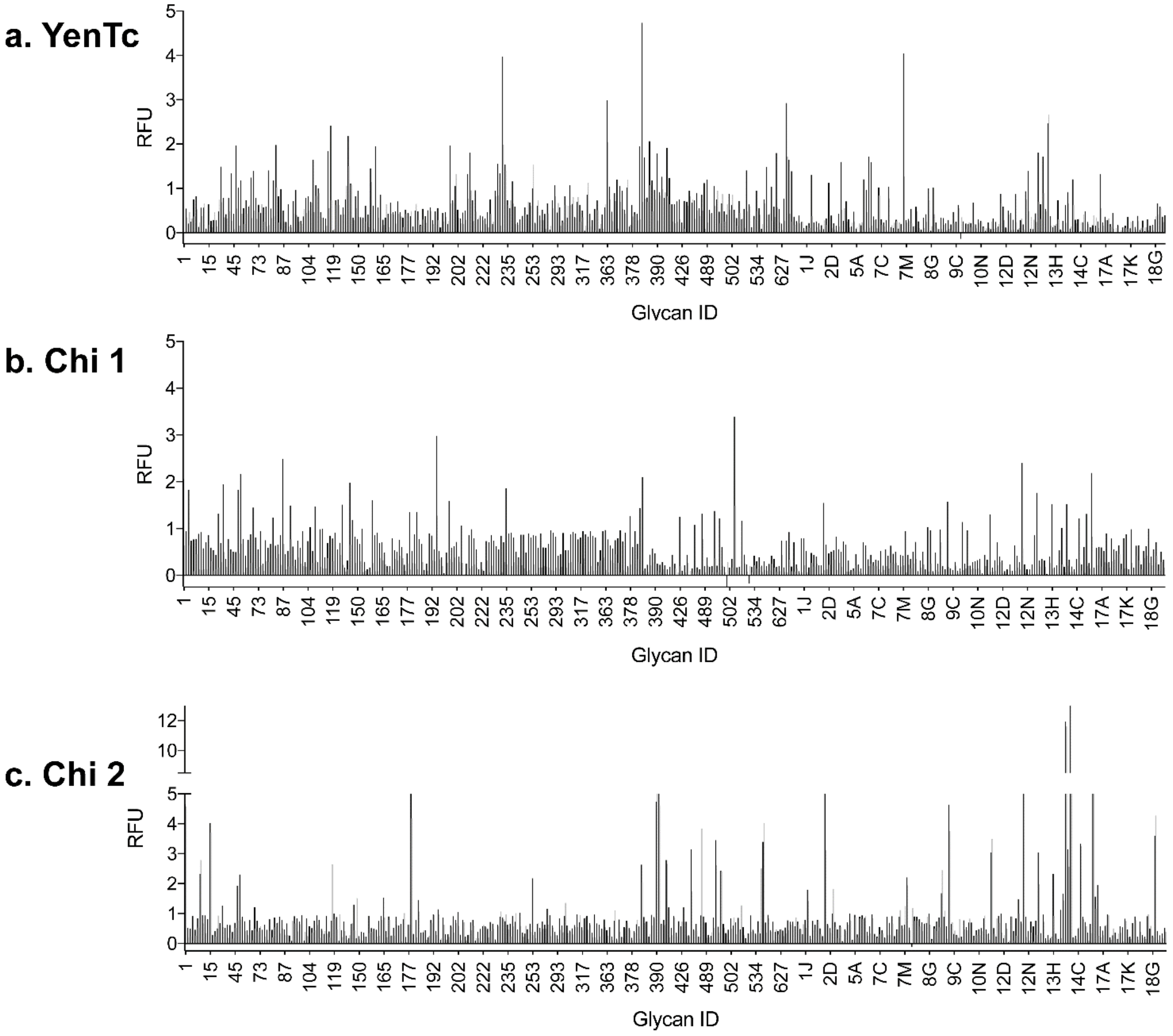

Supplementary Figure 11. Raw fluorescence reads from glycan array analysis. Data shown are the average of triplicate assays performed using labelled YenTc **(a)**, Chi1 **(b)** or Chi2 **(c)**. For each glycan, the black bar represents the average RFU while the grey bar represents the standard deviation from the three replicates. Binding was only classified as positive if three conditions were met: the response in relative fluorescence units (RFU) was significantly higher than the background ($P<0.005$); the response was higher than the adjusted background (defined as the average of negative control spots plus 3 standard deviations); and the response was positive in all three independent replicates. Glycan IDs are documented in Supplementary Data 1. Further details of the assay are included in Methods.

Supplementary References

1. Croll, T.I. ISOLDE: a physically-realistic environment for model building into low-resolution electron density maps. *Acta Cryst D* **74**, 519-530 (2018).
2. Lang, Alexander E., Konukiewitz, J., Aktories, K. & Benz, R. TcdA1 of Photorhabdus luminescens: Electrophysiological Analysis of Pore Formation and Effector Binding. *Biophys J* **105**, 376-384 (2013).
