# Supplementary material for: Cryo-EM structures of the pore-forming A subunit from the Yersinia entomophaga ABC toxin
Source: Nat Commun. 2019 Apr 26;10:1952. doi: 10.1038/s41467-019-09890-8 (PMC6486591; doi:10.1038/s41467-019-09890-8)
Supplement: Supplementary file 3 — Description of Additional Supplementary Files [file 41467_2019_9890_MOESM3_ESM.pdf]

Description of Additional Supplementary Files

File Name: Supplementary Data 1
Description: List of the 423 unique glycans, grouped in 15 distinct classes, printed on the array for each screen.

File Name: Supplementary Movie 1
Description: Visualisation of structural transitions between the pre-pore and pore configuration of YenTcA.
