# Supplementary material for: Cryo-EM structures of the pore-forming A subunit from the Yersinia entomophaga ABC toxin
Source: Nat Commun. 2019 Apr 26;10:1952. doi: 10.1038/s41467-019-09890-8 (PMC6486591; doi:10.1038/s41467-019-09890-8)
Supplement: Supplementary file 4 — Supplementary Data 1 [file 41467_2019_9890_MOESM4_ESM.pdf]

Supplementary Data 1. List of the 423 unique glycans, grouped in 15 distinct classes, printed on the array for each screen. For each glycan, the library ID, full name and molecular weight are shown.

**ID**	**Name**	**MW** (g/mol)
**Monosaccharides**		
1	Fucα-sp3	221.3
3	Galβ-sp3	237.3
4	GalNAcα-sp0	308.3
5	GalNAcα-sp3	278.3
6	GalNAcβ-sp3	278.3
7	Glcα-sp3	237.3
9	Glcβ-sp3	237.3
10	GlcNAcβ-sp3	278.3
14	GlcN(Gc)β-sp4	293.3
15	$HOCH_2(HOCH)_4CH_2NH_2$	181.2
19	ManNAcβ-sp4	277.3
20	Rhaα-sp3	221.3
22	GlcNAcβ-sp4	277.3
37	3-O-Su-Galβ-sp3	317.3
38	3-O-Su-GalNAcα-sp3	358.4
43	6-O-Su-GlcNAcβ-sp3	358.4
44	GlcAα-sp3	251.2
45	GlcAβ-sp3	251.2
46	6-H_2PO_3Glcβ-sp4	316.2
47	6-H_2PO_3Manα-sp3	339.2
48	Neu5Acα-sp3	366.4
49	Neu5Acα-sp9	471.5
52	Neu5Gcα-sp3	382.4
54	9-NAc-Neu5Acα-sp3	407.4
55	3-O-Su-GlcNAcβ-sp3	358.4
**Disaccharides**		
71	Fucα1-2Galβ-sp3	383.4
72	Fucα1-3GlcNAcβ-sp3	424.5
73	Fucα1-4GlcNAcβ-sp3	424.5
75	Galα1-2Galβ-sp3	399.4
77	Galα1-3GalNAcβ-sp3	440.5
78	Galα1-3GalNAcα-sp3	440.5
80	Galα1-3GlcNAcβ-sp3	440.5
81	Galα1-4GlcNAcβ-sp3	440.5
83	Galα1-6Glcβ-sp4	398.4
84	Galβ1-2Galβ-sp3	399.4
85	Galβ1-3GlcNaAcβ-sp3	440.5
87	Galβ1-3Galβ-sp3	399.4
88	Galβ1-3GalNAcβ-sp3	440.5
89	Galβ1-3GalNAcα-sp3	440.5
93	Galβ1-4Glcβ-sp4	398.4
94	Galβ1-4Galβ-sp4	398.4
97	Galβ1-4GlcNAcβ-sp3	440.5
100	Galβ1-6Galβ-sp4	398.4

101	GalNAcα1-3GalNAcβ-sp3	481.5
103	GalNAcα1-3GalNAcα-sp3	481.5
104	GalNAcβ1-3Galβ-sp3	440.5
106	GalNAcβ1-4GlcNAcβ-sp3	481.5
110	Glcα1-4Glcβ-sp3	399.4
111	Glcβ1-4Glcβ-sp4	398.4
112	Glcβ1-6Glcβ-sp4	398.4
113	GlcNAcβ1-3GalNAcα-sp3	481.5
114	GlcNAcβ1-3Manβ-sp4	439.4
115	GlcNAcβ1-4GlcNAcβ-Asn	538.5
117	GlcNAcβ1-4GlcNAcβ-sp4	480.5
118	GlcNAcβ1-6GalNAcα-sp3	481.5
119	Manα1-2Manβ-sp4	398.4
120	Manα1-3Manβ-sp4	398.4
121	Manα1-4Manβ-sp4	398.4
122	Manα1-6Manβ-sp4	398.4
123	Manβ1-4GlcNAcβ-sp4	439.4
124	Manα1-2Manα-sp4	398.4
145	Galβ1-3(6-O-Su)GlcNAcβ-sp3	520.5
146	Galβ1-4(6-O-Su)Glcβ-sp2	465.5
147	Galβ1-4(6-O-Su)GlcNAcβ-sp3	520.5
149	GlcNAcβ1-4(6-O-Su)GlcNAcβ-sp2	547.5
150	3-O-Su-Galβ1-3GalNAcα-sp3	520.5
151	6-O-Su-Galβ1-3GalNAcα-sp3	520.5
152	3-O-Su-Galβ1-4Glcβ-sp2	465.3
153	6-O-Su-Galβ1-4Glcβ-sp2	465.3
155	3-O-Su-Galβ1-3GlcNAcβ-sp3	520.5
157	3-O-Su-Galβ1-4GlcNAcβ-sp3	520.5
159	4-O-Su-Galβ1-4GlcNAcβ-sp3	520.5
161	6-O-Su-Galβ1-3GlcNAcβ-sp3	520.5
163	6-O-Su-Galβ1-4GlcNAcβ-sp3	520.5
164	GlcAβ1-3GlcNAcβ-sp3	454.4
165	GlcAβ1-3Galβ-sp3	413.4
166	GlcAβ1-6Galβ-sp3	413.4
167	GlcNAcβ1-4-[HOOC(CH_3)CH]-3-O-GlcNAcβ-sp4	522.5
168	GlcNAcβ1-4-[HOOC(CH_3)CH]-3-O-GlcNAcβ-L-Ala-D-i-Gln-L-Lys	823.9
169	Neu5Acα2-3Galβ-sp3	528.5
170	Neu5Acα2-6Galβ-sp3	528.5
171	Neu5Acα2-3GalNAcα-sp3	569.6
172	Neu5Acα2-6GalNAcα-sp3	569.6
174	Neu5Gcα2-6GalNAcα-sp3	585.6
176	3-O-Su-Galβ1-4(6-O-Su)Glcβ-sp2	567.5
177	3-O-Su-Galβ1-4(6-O-Su)GlcNAcβ-sp2	622.6
178	6-O-Su-Galβ1-4(6-O-Su)Glcβ-sp2	567.5
179	6-O-Su-Galβ1-3(6-O-Su)GlcNAcβ-sp2	608.5
180	6-O-Su-Galβ1-4(6-O-Su)GlcNAcβ-sp2	608.5
181	3,4-O-Su2-Galβ1-4GlcNAcβ-sp3	622.6
182	3,6-O-Su2-Galβ1-4GlcNAcβ-sp2	608.5
183	4,6-O-Su2-Galβ1-4GlcNAcβ-sp2	608.5

184	4,6-O-Su2-Galβ1-4GlcNAcβ-sp3	622.6
186	Neu5Acα2-8Neu5Acα2-sp3	679.6
189	3,6-O-Su2-Galβ1-4(6-O-Su)GlcNAcβ-sp2	710.6
192	GalNAcβ1-4(6-O-Su)GlcNAcβ-sp3	561.5
193	3-O-Su-GalNAcβ1-4GlcNAcβ-sp3	561.5
194	6-O-Su-GalNAcβ1-4GlcNAcβ-sp3	561.5
195	6-O-Su-GalNAcβ1-4-(3-O-Su)GlcNAcβ-sp3	603.5
196	3-O-Su-GalNAcβ1-4(3-O-Su)-GlcNAcβ-sp3	663.5
197	3,6-O-Su2-GalNAcβ1-4GlcNAcβ-sp3	663.5
198	4,6-O-Su2-GalNAcβ1-4GlcNAcβ-sp3	663.5
199	4,6-O-Su2-GalNAcβ1-4-(3-O-Ac)GlcNAcβ-sp3	705.5
200	4-O-Su-GalNAcβ1-4GlcNAcβ-sp3	561.5
201	3,4-O-Su2-Galβ1-4GlcNAcβ-sp3	663.5
202	6-O-Su-GalNAcβ1-4(6-O-Su)GlcNAcβ-sp3	663.5
203	Galβ1-4(6-O-Su)GlcNAcβ-sp2	506.5
204	4-O-Su-GalNAcβ1-4GlcNAcβ-sp2	547.5
205	Neu5Acα2-6GalNAcβ-sp3	569.6
206	Neu5Gcα2-3Gal-sp3	544.5
**Trisaccharides**		
215	Fucα1-2Galβ1-3GlcNAcβ-sp3	586.6
216	Fucα1-2Galβ1-4GlcNAcβ-sp3	586.6
217	Fucα1-2Galβ1-3GalNAcα-sp3	586.6
219	Fucα1-2Galβ1-4Glcβ-sp4	544.5
220	Galα1-3Galβ1-4Glcβ-sp2	547.5
222	Galα1-3Galβ1-4GlcNAcβ-sp3	602.6
224	Galα1-4Galβ1-4Glcβ-sp3	561.5
225	Galα1-4Galβ1-4GlcNAc-sp2	588.6
226	Fucα1-2(Galα1-3)Galβ-sp3	545.5
228	Galβ1-2Galα1-4GlcNAcβ-sp4	601.6
229	Galβ1-3Galβ1-4GlcNAcβ-sp4	601.6
231	Galβ1-4GlcNAcβ1-3GalNAcα-sp3	643.6
232	Galβ1-4GlcNAcβ1-6GalNAcα-sp3	643.6
233	Galβ1-3(Fucα1-4)GlcNAcβ-sp3	586.6
234	Fucα1-3(Galβ1-4)GlcNAcβ-sp3	586.6
235	Fucα1-2(GalNAcα1-3)Galβ-sp3	586.6
238	GalNAcβ1-4Galβ1-4Glcβ-sp3	602.6
240	(Glcα1-4)$_3$β-sp4	560.5
241	(Glcα1-6)$_3$β-sp4	560.5
246	GlcNAcβ1-2Galβ1-3GalNAcα-sp3	643.6
247	GlcNAcβ1-3Galβ1-3GalNAcα-sp3	643.6
248	GlcNAcβ1-3Galβ1-4Glcβ-sp2	588.6
250	GlcNAcβ1-3Galβ1-4GlcNAcβ-sp3	643.6
251	GlcNAcβ1-4Galβ1-4GlcNAcβ-sp2	629.6
252	GlcNAcβ1-4GlcNAcβ1-4GlcNAcβ-sp4	683.6
253	GlcNAcβ1-6Galβ1-4GlcNAcβ-sp2	629.6
254	Galβ1-3(GlcNAcβ1-6)GalNAcα-sp3	643.6
255	GlcNAcβ1-3(GlcNAcβ1-6)GalNAcα-sp3	684.7
258	Manα1-3(Manα1-6)Manβ-sp4	560.5
262	Galβ1-3GalNAcβ1-3Gal-sp4	601.6

264	Galβ1-4Galβ1-4GlcNAc-sp3	602.6
287	3-O-Su-Galβ1-3(Fucα1-4)GlcNAcβ-sp3	666.7
288	Fucα1-3(3-O-Su-Galβ1-4)GlcNAcβ-sp3	666.7
289	Galα1-3(Neu5Acα2-6)GalNAcα-sp3	731.7
290	Galβ1-3(Neu5Acα2-6) GalNAcα-sp3	731.7
292	Neu5Acα2-3Galβ1-3GalNAcα-sp3	731.7
293	Neu5Acα2-3Galβ1-4Glcβ-sp3	690.7
294	Neu5Acα2-3Galβ1-4Glcβ-sp4	689.6
295	Neu5Acα2-6Galβ1-4Glcβ-sp2	676.6
298	Neu5Acα2-3Galβ1-4GlcNAcβ-sp3	731.7
299	Neu5Acα2-3Galβ1-3GlcNAcβ-sp3	731.7
300	Neu5Acα2-6Galβ1-4GlcNAcβ-sp3	731.7
303	Neu5Gcα2-3Galβ1-4GlcNAcβ-sp3	747.7
304	Neu5Gcα2-6Galβ1-4GlcNAcβ-sp3	747.7
306	9-NAc-Neu5Acα2-6Galβ1-4GlcNAcβ-sp3	788.8
315	Neu5Acα2-3Galβ1-4-(6-O-Su)GlcNAcβ-sp3	833.8
317	Neu5Acα2-3Galβ1-3-(6-O-Su)GalNAcβ-sp3	833.8
318	Neu5Acα2-6Galβ1-4-(6-O-Su)GlcNAcβ-sp3	833.8
319	Neu5Acα2-3-(6-O-Su)Galβ1-4GlcNAcβ-sp3	833.8
321	(Neu5Acα2-8)$_3$-sp3	992.9
323	Neu5Acα2-6Galβ1-3GlcNAc-sp3	731.7
324	Neu5Acα2-6Galβ1-3(6-O-Su)GlcNAc-sp3	883.8
331	Neu5Gcα2-3Galβ1-3GlcNAcβ-sp3	747.7
**Tetrasaccharides**		
359	Fucα1-2(Galα1-3)Galβ1-3GlcNAcβ-sp3	748.7
360	Fucα1-2(Galα1-3)Galβ1-4GlcNAcβ-sp3	748.7
362	Fucα1-2(Galα1-3)Galβ1-3GalNAcα-sp3	748.7
363	Fucα1-2(Galα1-3)Galβ1-3GalNAcβ-sp3	748.7
364	Fucα1-3(Galα1-3Galβ1-4)GlcNAcβ-sp3	748.7
366	Fucα1-2(GalNAcα1-3)Galβ1-3GlcNAcβ-sp3	789.8
368	Fucα1-2(GalNAcα1-3)Galβ1-4GlcNAcβ-sp3	789.8
371	Fucα1-2Galβ1-3(Fucα1-4)GlcNAcβ-sp3	732.7
372	Fucα1-3(Fucα1-2Galβ1-4)GlcNAcβ-sp3	732.7
373	Galα1-3Galβ1-4GlcNAcβ1-3Galβ-sp3	764.7
375	Galα1-4GlcNAcβ1-3Galβ1-4GlcNAcβ-sp3	805.8
376	Galβ1-3GlcNAcβ1-3Galβ1-4Glcβ-sp4	763.7
377	Galβ1-3GlcNAcβ1-3Galβ1-3GlcNAcβ-sp2	791.8
378	Galβ1-3GlcNAcα1-3Galβ1-4GlcNAcβ-sp3	805.8
379	Galβ1-3GlcNAcβ1-3Galβ1-4GlcNAcβ-sp3	805.8
380	Galβ1-3GlcNAcα1-6Galβ1-4GlcNAcβ-sp2	791.8
381	Galβ1-3GlcNAcβ1-6Galβ1-4GlcNAcβ-sp2	791.8
382	Galβ1-3GalNAcβ1-4Galβ1-4Glcβ-sp3	764.7
383	Galβ1-4GlcNAcβ1-3Galβ1-4Glcβ-sp2	763.7
385	Galβ1-4GlcNAcβ1-3Galβ1-4GlcNAcβ-sp3	805.8
387	Galβ1-4GlcNAcβ1-6Galβ1-4GlcNAcβ-sp2	791.7
388	Galβ1-3(Galβ1-4GlcNAcβ1-6)GalNAcα-sp3	805.8
389	GalNAcβ1-3Galα1-4Galβ1-4Glcβ-sp3	764.7
390	(Glcα1-4)$_4$β-sp4	722.7

391	(Glcα1-6)$_4$β-sp4	722.7
392	Fucα1-2(GalNAcα1-3)Galβ1-3GalNAcα-sp3	789.8
395	GlcNAcβ1-3(GlcNAcβ1-6)Galβ1-4GlcNAcβ-sp3	832.8
401	Galβ1-3GlcNAcβ1-3Galβ1-3GlcNAcβ-sp3	805.8
419	3-O-SuGalβ1-4GlcNAcβ1-3Galβ1-4GlcNAcβ-sp3	907.8
420	4-O-SuGalβ1-4GlcNAcβ1-3Galβ1-4GlcNAcβ-sp3	907.8
421	Neu5Acα2-3(GalNAcβ1-4)Galβ1-4Glcβ-sp2	879.8
422	Neu5Acα2-3Galβ1-4GlcNAcβ1-3Galβ-sp3	893.9
423	Fucα1-3(Neu5Acα2-3Galβ1-4)GlcNAcβ-sp3	877.9
426	Neu5Acα2-3Galβ1-3(Fucα1-4)GlcNAcβ-sp3	877.9
428	Fucα1-3(Neu5Acα2-3Galβ1-4)6-O-Su-GlcNAcβ-sp3	979.9
429	Fucα1-3(Neu5Acα2-3(6-O-Su)Galβ1-4)GlcNAcβ-sp3	979.9
433	Neu5Acα2-3Galβ1-3(Neu5Acα2-6)GalNAcα-sp3	1044.9
434	Neu5Acα2-8Neu5Acα2-3Galβ1-4Glcβ-sp4	1002.9
**Penta-Nonasaccharides**		
479	Fucα1-2Galβ1-3GlcNAcβ1-3Galβ1-4Glcβ-sp4	909.9
480	Fucα1-2Galβ1-3GlcNAcβ1-3Galβ1-4GlcNAcβ-sp2	937.9
481	Galα1-3Galβ1-4GlcNAcβ1-3Galβ1-4Glcβ-sp4	925.8
483	Fucα1-3(Fucα1-2(Galα1-3)Galβ1-4)GlcNAcβ-sp3	894.9
488	Galβ1-4GlcNAcβ1-3(Galβ1-4GlcNAcβ1-6)GalNAcα-sp3	1008.9
489	Galβ1-4GlcNAcβ1-3(GlcNAcβ1-6)Galβ1-4GlcNAc-sp2	994.9
490	GlcNAcβ1-3(Galβ1-4GlcNAcβ1-6)Galβ1-4GlcNAcβ-sp2	994.9
492	(Glcα1-6)$_5$β-sp4	884.8
493	(GlcNAcβ1-4)$_5$β-sp4	1090
495	Manα1-3(Manα1-3(Manα1-6)Manα1-6)Manβ-sp4	966.9
496	Fucα1-2Galβ1-3(Fucα1-4)GlcNAcβ1-3Galβ1-4Glcβ-sp4	1056
497	Fucα1-3(Fucα1-2Galβ1-4)GlcNAcβ1-3Galβ1-4Glcβ-sp4	1056.0
498	(Galβ1-4GlcNAcβ1-3)$_3$-sp3	1171.1
499	Galβ1-4GlcNAcβ1-3(Galβ1-4GlcNAcβ1-6)Galβ1-4GlcNAc- sp2	1157.1
501	Galβ1-3GalNAcβ1-3Galα1-4Galβ1-4Glcβ-sp4	925.8
502	(Glcα1-6)$_6$β-sp4	1046.9
503	(GlcNAcβ1-4)$_6$β-sp4	1293.3
504	(A-GN-M)2-3,6-M-GN-GNβ-sp4	1697.6
505	(GN-M)2-3,6-M-GN-GNβ-sp4	1373.3
527	Neu5Acα2-3Galβ1-4GlcNAcβ1-3Galβ1-4GlcNAcβ-sp2	1083.0
528	Fucα1-3(Neu5Acα2-3 Galβ1-4)GlcNAcβ1-3Galβ-sp3	1040.0
529	Neu5Acα2-6(Galβ1-3)GlcNAcβ1-3Galβ1-4Glcβ-sp4	1055.0
531	GalNAcβ1-4(Neu5Acα2-8Neu5Acα2-3)Galβ1-4Glc-sp2	1193.1
532	Neu5Acα2-8Neu5Acα2-8Neu5Acα2-3Galβ1-4Glc-sp2	1303.1
533	(Neu5Acα2-8)$_2$Neu5Acα2-3(GalNAcβ1-4)Galβ1-4Glc-sp2	1506.3
534	Neu5Acα2-3Galβ1-4GlcNAcβ1-3Galβ1-4GlcNAcβ-sp3	1097.1
536	Neu5Acα2-3Galβ1-3GlcNAcβ1-3Galβ1-4Glcβ-sp4	1055.0
537	Neu5Acα2-3Galβ1-4GlcNAcβ1-3Galβ1-4Glcβ-sp4	1055.0
538	Le^x1-6'(Le^c1-3')Lac-sp4	1389.2
539	LacNAc1-6'(Led1-3')Lac-sp4	1389.2
540	Le^x1-6'(6'SLN1-3')Lac-sp4	1566.5
541	Le^x1-6'(Le^d1-3')Lac-sp4	1535.4
542	Le^cLe^x1-6'(Le^c1-3')Lac-sp4	1754.6

543	Le^x1-6'(Le^b1-3')Lac-sp4	1681.5
**Higher Oligosaccharides**		
625	(GlcAβ1-4GlcNAcβ1-3)$_8$-NH_2-ol	3207.5
627	(Sia2-6A-GN-M)2-3,6-M-GN-GNβ-sp4	2302.1
**Terminal Galactose**		
1A	Galβ1-3GlcNAc	383.35
1B	Galβ1-4GlcNAc	383.35
1C	Galβ1-4Gal	342.3
1D	Galβ1-6GlcNAc	383.3
1E	Galβ1-3GalNAc	383.3
1F	Galβ1-3GalNAcβ1-4Galβ1-4Glc	707.6
1G	Galβ1-3GlcNAcβ1-3Galβ1-4Glc	707.6
1H	Galβ1-4GlcNAcβ1-3Galβ1-4Glc	707.6
1I	Galβ1-4GlcNAcβ1-6(Galβ1-4GlcNAcβ1-3)Galβ1-4Glc	1072.9
1J	Galβ1-4GlcNAcβ1-6(Galβ1-3GlcNAcβ1-3)Galβ1-4Glc	1072.9
1K	Galα1-4Galβ1-4Glc	504.4
1L	GalNAcα1-O-Ser	308.2
1M	Galβ1-3GalNAcα1-O-Ser	470.4
1N	Galα1-3Gal	342.2
1O	Galα1-3Galβ1-4GlcNAc	545.4
1P	Galα1-3Galβ1-4Glc	504.4
2A	Galα1-3Galβ1-4Galα1-3Gal	666.5
2B	Galβ1-6Gal	342.3
2C	GalNAcβ1-3Gal	383.3
2D	GalNAcβ1-4Gal	383.3
2E	Galα1-4Galβ1-4GlcNAc	545.4
2F	GalNAcα1-3Galβ1-4Glc	545.4
2G	Galβ1-3GlcNAc β1-3Galβ1-4GlcNAc β1-6(Galβ1- 3GlcNAc β1-3)Galβ1-4Glc	1438.3
2H	Galβ1-3GlcNAc β1-3Galβ1-4GlcNAc β1-3Galβ1-4Glc	1072.9
18B	Galβ1-3GalNAcβ1-3Galα1-4Galβ1-4Glc	869.7
18C	Galβ1-3GalNAcβ1-3Gal	545.4
18L	Galβ1-4Glc	342.3
18M	Galβ1-4Gal	360.3
18N	Galβ1-6Gal	342.3
**Terminal GlcNAc**		
4A	GlcNAcβ1-4GlcNAc	424.4
4B	GlcNAcβ1-4GlcNAcβ1-4GlcNAc	627.5
4C	GlcNAcβ1-4GlcNAcβ1-4GlcNAcβ1-4GlcNAc	830.7
4D	GlcNAcβ1-4GlcNAcβ1-4GlcNAcβ1-4GlcNAcβ1- 4GlcNAcβ1-4GlcNAc	1237.1
4E	Bacterial cell wall muramyl discaccharide	496.4
4F	GlcNAcβ1-4GlcNAcβ1-4GlcNAcβ1-4GlcNAcβ1-4GlcNAc	1033.9
18G	6-O-Su-GlcNAc	323.2
18H	GlcNAc	221.2
**Mannosyl-containing Glycans**		
5A	GlcNAcβ1-2Man	383.3
5B	GlcNAcβ1-2Manα1-6(GlcNAcβ1-2Manα1-3)Man	910.8
5C	Manα1-2Man	342.3

5D	Manα1-3Man	342.3
5E	Manα1-4Man	342.3
5F	Manα1-6Man	342.3
5G	Manα1-6(Manα1-3)Man	504.4
5H	Manα1-6(Manα1-3)Manα1-6(Manα1-3)Man	828.7
**Fucosylated Glycans**		
7A	Fucα1-2Galβ1-3GlcNAcβ1-3Galβ1-4Glc	853.7
7B	Galβ1-3(Fucα1-4)GlcNAcβ1-3Galβ1-4Glc	853.7
7C	Galβ1-4(Fucα1-3)GlcNAcβ1-3Galβ1-4Glc	853.7
7D	Fucα1-2Galβ1-3(Fucα1-4)GlcNAcβ1-3Galβ1-4Glc	999.9
7E	Galβ1-3(Fucα1-4)GlcNAcβ1-3Galβ1-4(Fucα1-3)Glc	999.9
7F	Fucα1-2Gal	326.3
7G	Fucα1-2Galβ1-4Glc	488.4
7H	Galβ1-4(Fucα1-3)Glc	488.4
7I	Galβ1-4(Fucα1-3)GlcNAc	529.4
7J	Galβ1-3(Fucα1-4)GlcNAc	529.4
7K	GalNAc α1-3(Fucα1-2)Gal	529.4
7L	Fucα1-2Galβ1-4(Fucα1-3)Glc	616.2
7M	Galβ1-3(Fucα1-2)Gal	488.4
7N	Fucα1-2Galβ1-4(Fucα1-3)GlcNAc	675.6
7O	Fucα1-2Galβ1-3GlcNAc	529.4
7P	Fucα1-2Galβ1-3(Fucα1-4)GlcNAc	675.6
8A	SO3-3Galβ1-3(Fucα1-4)GlcNAc	631.5
8B	SO3-3Galβ1-4(Fucα1-3)GlcNAc	631.5
8C	Galβ1-3GlcNAc β1-3Galβ1-4(Fucα1-3)GlcNAc β1-3Galβ1- 4Glc	1219.1
8D	Galβ1-4(Fucα1-3)GlcNAc β1-6(Galβ1-3GlcNAc β1-3)Galβ1- 4Glc	1219.1
8E	Galβ1-4(Fucα1-3)GlcNAc β1-6(Fucα1-2Galβ1-3GlcNAc β1- 3)Galβ1-4Glc	1365.2
8F	Galβ1-4(Fucα1-3)GlcNAc β1-6(Fucα1-2Galβ1-3(Fucα1- 4)GlcNAc β1-3)Galβ1-4Glc	1511.3
8G	Galβ1-4GlcNAcβ1-3Galβ1-4(Fucα1-3)Glc	853.7
8H	Fucα1-2Galβ1-4(Fucα1-3)GlcNAcβ1-3Galβ1-4Glc	999.9
8I	Fucα1-3Galβ1-4GlcNAcβ1-3Galβ1-4(Fucα1-3)Glc	999.9
8J	Fucα1-2Galβ1-4(Fucα1-3)GlcNAcβ1-3(Fucα1-2)Galβ1-4Glc	1146.0
8K	Galβ1-4(Fucα1-3)GlcNAcβ1-6(Galβ1-4GlcNAcβ1-3)Galβ1-4	1219.1
8L	Galβ1-4(Fucα1-3)GlcNAcβ1-6(Galβ1-4(Fucα1-3)GlcNAcβ1- 3)Galβ1-4Glc	1365.2
8M	Fucα1-2Galβ1-4(Fucα1-3)GlcNAcβ1-6(Galβ1-4GlcNAcβ1- 3)Galβ1-4Glc	1365.2
8N	Galβ1-3GlcNAcβ1-3Galβ1-4(Fucα1-3)GlcNAcβ1-6(Galβ1- 3GlcNAcβ1-3)Galβ1-4Glc	1584.4
8O	Fucα1-2Galβ1-3GlcNAcβ1-3Galβb1-4(Fucα1-3)GlcNAcβ1- 6(Galβ1-3GlcNAcβ1-3)Galβ1-4Glc	1876.7
8P	GalNAcα1-3(Fucα1-2)Galβ1-4GalNAc	732.6
9A	Galα1-3(Fucα1-2)Galβ1-4(Fucα1-3)Glc	796.7
9B	Galβ1-4GlcNAc β1-6(Fucα1-2Galβ1-3GlcNAc β1-3)Galβ1- 4Glc	1219.1
18D	Galα1-3(Fucα1-2)Galβ1-4Glc	650.5
18E	GalNAcα1-3(Fucα1-2)Galβ1-4(Fucα1-3)Glc	837.7
19J	Galβ1-4(Fucα1-3)GlcNAcβ1-3Gal	691.6
19L	Fucα1-2Galβ1-4(Fucα1-3)GlcNAcβ1-3Gal	837.7
19M	Galβ1-3(Fucα1-4)GlcNAcβ1-3Gal	691.6

19N	Fucα1-2Galβ1-3(Fucα1-4)GlcNAcβ1-3Gal	837.7
**Sialylated Glycans**		
10A	Neu5Acα2-3Galβ1-3(Fucα1-4)GlcNAc	820.7
10B	Neu5Acα2-3Galβ1-4(Fucα1-3)GlcNAc	820.7
10C	Neu5Acα2-3Galβ1-3GlcNAcβ1-3Galβ1-4Glc	818.2
10D	Galβ1-4(Fucα1-3)GlcNAc β1-6(Neu5Acα2-6Galβ1- 4Glc*NAc* β1-3)Galβ1-4Glc	1510.3
10E	Neu5Acα2-3Galβ1-3(Neu5Acα2-6)GalNAc	965.8
10H	Neu5Acα2-6Galβ1-3GlcNAcβ1-3Galβ1-4(Fucα1-3)Glc	1145.0
10I	Galβ1-3GlcNAcβ1-3(Neu5Acα2-6Galβ1-4GlcNAcβ1- 6)Galβ1-4Glc	1364.2
10J	Neu5Acα2-6Galβ1-3GlcNAcβ1-3(Galβ1-4GlcNAcβ1- 6)Galβ1-4Glc	1364.2
10K	Neu5Acα2-3Galβ1-4GlcNAc	674.6
10L	Neu5Acα2-6Galβ1-4GlcNAc	674.6
10M	Neu5Acα2-3Galβ1-3GlcNAcβ1-3Galβ1-4Glc	998.8
10N	Galβ1-3(Neu5Acα2-6)GlcNAc β1-3Galβ1-4Glc	998.8
10O	Neu5Acα2-6Galβ1-4GlcNAc β1-3Galβ1-4Glc	998.8
10P	Neu5Acα2-3Galβ1-3(Neu5Acα2-6)GlcNA*c* β1-3Galβ1-4Glc	1290.1
11A	Neu5Acα2-3Galβ1-4Glc	633.5
11B	Neu5Acα2-6Galβ1-4Glc	633.5
11C	(Neu5Acα2-8Neu5Ac)$_n$ *(n<50)*	
18A	Neu5Acα2-3Galβ1-4GlcNAcβ1-3Galβ1-4Glc	1020.8
18K	9-NAc-Neu5Ac	351.3
18O	Neu5Gc	325.2
19K	Neu5Acα2-3Galβ1-4(Fucα1-3)GlcNAcβ1-3Gal	1004.8
**Carageenan & Glycosaminoglycans**		
12A	Neocarratetraose-41, 3-di-O-sulphate	834.6
12B	Neocarratetraose-41-O-sulphate	732.5
12C	Neocarrahexaose-24,41, 3, 5-tetra-O-sulphate	1344.9
12D	Neocarrahexaose-41, 3, 5-tri-O-sulphate	1242.9
12E	Neocarraoctaose-41, 3, 5, 7-tetra-O-sulphate	1651.2
12F	Neocarradecaose-41, 3, 5, 7, 9-penta-O-sulphate	2059.5
12G	ΔUA-2S-GlcNS-6S	665.4
12H	ΔUA-GlucNS-6S	563.3
12I	ΔUA-2S-GlucNS	563.3
12J	ΔUA-2S-GlcNAc-6S	605.3
12K	ΔUA-GlcNAc-6S	503.3
12L	ΔUA-2S-GlcNAc	503.3
12M	ΔUA-GlcNAc	401.3
12N	ΔUA-GalNAc-4S (Delta Di-4S)	503.3
12O	ΔUA-GalNAc-6S (Delta Di-6S)	503.3
12P	ΔUA-GalNAc-4S,6S (Delta Di-disE)	605.3
13A	ΔUA-2S-GalNAc-4S (Delta Di-disB)	605.3
13B	ΔUA-2S-GalNAc-6S (Delta Di-disD)	605.3
13C	ΔUA-2S-GalNAc-4S-6S (Delta Di-tisS)	707.4
13D	ΔUA-2S-GalNAc-6S (Delta Di-UA2S)	503.3
13E	ΔUA-GlcNAc (Delta Di-HA)	401.3
13F	(GlcAβ1-3GlcNAc β1-4)$_n$ *(n=4)*	
13G	(GlcAβ1-3GlcNAc β1-4)$_n$ *(n=8)*	
13H	(GlcAβ1-3GlcNAc β1-4)$_n$ *(n=10)*	

13I	(GlcAβ1-3GlcNAc β1-4)$_n$ *(n=12)*	
13J	(GlcA/IdoAα/β1-4GlcNAc α1-4)$_n$ *(n=200)*	
13K	(GlcA/IdoAβ1-3(±4/6S)GalNAc β1-4)$_n$ *(n<250)*	
13L	((±2S)GlcA/IdoAα/b1-3(±4S)GalNAc β1-4)$_n$ *(n<250)*	
13M	(GlcA/IdoAβ1-3(±6S)GalNAc β1-4)$_n$ *(n<250)*	
13N	HA - 4	775
130	HA - 6	1155.6
13P	HA - 8	1534.7
14A	HA 10	1913.8
14B	HA-12	2293.4
14C	HA-14	2672.5
14D	HA-16	
14E	HA 30000 Da	
14F	HA 107000 Da	
14G	HA 190000 Da	
14H	HA 220000 Da	
14I	HA 1600000 Da	
14J	Heparin sulfate	
14K	β1-3Glucan	
14L	Chondroitin disaccharide Δdi-OS, sodium salt	401.3
14M	ΔUA→2S-GlcN-6S	541.3
14N	ΔUA→GlcN-6S	439.3
14O	ΔUA→2S-GlcN	439.3
14P	ΔUA→GlcN	337.2
18I	GlcA	194.1
**Terminal Glucose Structures**		
18J	6-O-(H_2PO_4)-Glc	260.1
19O	Glcα1-4Glcα1-4Glc	504.4
19P	Glcα1-4Glcα1-4Glcα1-4Glc	666.5
**Ganglioside Structures**		
17A	GalNAcβ1-4Galβ1-4Glc	545.4
17B	Galβ1-3GalNAcβ1-4Galβ1-4Glc	707.6
17C	Galβ1-3GalNAcβ1-4(Neu5Acα2-8Neu5Acα2-8 Neu5Acα2- 3)Galβ1-4Glc	1647.3
17D	Neu5Acα2-8Neu5Acα2-3Galβ1-3GalNAcβ1-4(Neu5Acα2- 3)Galβ1-4Glc	1647.3
17E	Galβ1-3GalNAcβ1-4(Neu5Acα2-8Neu5Acα2-3)Galβ1-4Glc	1334.0
17F	Neu5Acα2-3Galβ1-3GalNAcβ1-4(Neu5Acα2-3)Galβ1-4Glc	1334.0
17G	Neu5Acα2-3Galβ1-3GalNAcβ1-4Galβ1-4Glc	1020.8
17H	Galβ1-3GalNAcβ1-4(Neu5Acα2-3)Galβ1-4Glc	1020.8
17I	Fucα1-2Galβ1-3GalNAcβ1-4(Neu5Acα2-3)Galβ1-4Glc	1168.0
17J	GalNAcβ1-4(Neu5Acα2-8Neu5Acα2-8Neu5Acα2-3)Galβ1- 4Glc	1485.1
17K	GalNAcβ1-4(Neu5Acα2-8Neu5Acα2-3)Galβ1-4Glc	1171.9
17L	GalNAcβ1-4(Neu5Acα2-3)Galβ1-4Glc	858.7
17M	Neu5Acα2-8Neu5Acβ2-8Neu5Acα2-3Galβ1-4Glc	1282.0
17N	Neu5Acα2-8Neu5Acα2-3Galβ1-4Glc	968.7
17O	Neu5Acα2-3Galβ1-4Glc	655.5
**Complex Type N-Glycans**		
19A	Galβ1-4GlcNAcβ1-2Manα1-3(Galβ1-4GlcNAcβ1-2Manα1- 6Man)β1-4GlcNAcβ1-4(Fucα1-6)GlcNAc	1787.0

19B	Galβ1-4GlcNAcβ1-2(Galβ1-4GlcNAcβ1-4)Manα1-3(Galβ1- 4GlcNAcβ1-2(Galβ1-4GlcNAcβ1-6)Manα1-6Man)β1- 4GlcNAcβ1-4GlcNAc	2372.0
19C	Neu5Acα2-6Galβ1-4GlcNAcβ1-2Manα1-3(Galβ1- 4GlcNAcβ1-2Manα1-6)Manβ1-4GlcNAcβ1-4GlcNAc	1932.0
19D	Neu5Acα2-6Galβ1-4GlcNAcβ1-2Manα1-3(Neu5Acα2- 6Galβ1-4GlcNAcβ1-2Manα1-6)Manβ1-4GlcNAcβ1- 4GlcNAc	2224.0
19E	Galβ1-4GlcNAcβ1-2Manα1-3(Galβ1-4GlcNAcβ1-2Manα1- 6)Manβ1-4GlcNAcβ1-4GlcNAc	1641.0
19F	Neu5Acα2-6Galβ1-4GlcNAcβ1-2Manα1-3(Neu5Acα2- 6Galβ1-4GlcNAcβ1-2Manα1-6)Manβ1-4GlcNAcβ1- 4(Fucα1-6)GlcNAc	2370.0
19G	Neu5Acα2-6Galβ1-4GlcNAcβ1-2(Neu5Acα2-6Galβ1- 4GlcNAcβ1-4)Manα1-3(Neu5Acα2-6Galβ1-4GlcNAcβ1- 2Manα1-6)Manβ1-4GlcNAcβ1-4GlcNAc	2880.5
19H	GlcNAcβ1-2(GlcNAcβ1-4)Manα1-3(GlcNAcβ1-2Manα1- 6)GlcNAcβ1-4Manβ1-4GlcNAcβ1-4GlcNAc	1723.5
